# Revisional surgery for malnutrition after SADI-S: prevalence, indications, techniques and outcomes

**DOI:** 10.1007/s13304-024-01900-9

**Published:** 2024-05-28

**Authors:** Andrés Sánchez-Pernaute, Bibiana Lasses, Leyre López Antoñanzas, Miguel Ángel Rubio, Clara Marcuello, Natalia Pérez Ferré, Antonio Torres, Elia Pérez-Aguirre

**Affiliations:** 1 Department of Surgery, Hospital Clínico San Carlos, Universidad Complutense de Madrid, Madrid, Spain; 2 Department of Endocrinology, Hospital Clínico San Carlos, Universidad Complutense de Madrid, Madrid, Spain

**Keywords:** SADIS, Malabsorption, Revisional surgery

## Abstract

SADIS with short common limb (< 250 cm) is a malabsorptive operation.

Reoperation is advised in patients requiring admission for severe malnutrition.

Elongation of the common channel is the preferred revisional technique

Introduction: Single-Anastomosis Duodeno-Ileal bypass with Sleeve gastrectomy (SADI-S) is a modification of the duodenal switch. Initial common channel’s length was 200, and after malnutrition was detected in some patients, it was elongated to 250 or 300 cm. The present study analyzes presentation and treatment of malnutrition after SADI-S. Materials: Three hundred and thirty-three consecutive patients undergoing SADI-S between May 2007 and February 2019 were included. The common limb length was 200 cm in 50 cases, 250 cm in 211, 300 in 71 and 350 in 1. Thirty-one patients were admitted for severe hypoalbuminemia and 17 patients were submitted to revisional surgery, and constitute the series of our study. Mean weight before reoperation was 57 kg and mean body mass index (BMI) was 21 kg/m^2^. Mean number of daily bowel movements was 5,6. Results: Mean time to reoperation was 56 months. The limb was found shorter than expected in 6 cases. Revisional surgery was conversion into a Roux en Y duodenal switch in 3 cases, elongation of the common limb in 11 patients, duodeno-duodenostomy in 1 and duodeno-jejunostomy to the first jejunal loop in 2. Mean weight regain was 14 kg, and mean final BMI 26 kg/m^2^. Daily bowel movements were reduced to 1,3. Factors related to hypoalbuminemia were hypertension, poor-controlled diabetes, shorter common limb and liver-test alterations. Conclusion: SADI-S is expected to be less malabsorptive than previous biliopancreatic diversions. However, caution must be taken with certain patients to avoid postoperative malnutrition. Adequate follow up with long-term supplementation is required.

## Introduction

Malnutrition is one of the most feared long-term complications of bariatric surgery. It has been historically related to malabsorptive operations, as jejunoileal bypass (JIB), biliopancreatic diversion (BPD) and duodenal switch (DS), although patients submitted to less aggressive techniques can also present some degrees of malnutrition. Single-Anastomosis Duodeno-Ileal bypass with Sleeve Gastrectomy (SADI-S) was introduced as a simpler alternative to the Duodenal Switch (DS) in which malabsorption was supposed to be milder because of the increase in the length of the common channel [1]. However, a high rate of nutritional problems presented in the first series of patients in which the common limb measured 200 cm. This led us to elongate the absorptive channel (250 to 300 cm) to ameliorate the malabsorptive effect of the operation [2]. The new longer common channel still showed good weight loss results in the long-term, whereas there was a reduction in the nutritional issues [3, 4]. Despite this greater absorption, some patients still present with hypoalbuminemia and other deficiencies [5], perhaps secondarily to a wrong selection, errors in the limb measurement or poor adherence to diet and supplementation. The management of malnourished patients can be difficult, and many times involves the revision of the technique. This seems to be easier in a one-limb operation when compared to Roux en Y configuration, but still the handling of a duodeno-intestinal anastomosis can be a concern for many surgeons.

In the present study, we review our experience on malnutrition after SADI-S and analyze the possible causes for this malnutrition, the markers that permit an early detection of these patients to avoid dreadful consequences, the current surgical solutions to improve their nutritional status and the outcomes after revisional surgery.

## Patients and methods

**Fig. 1:**
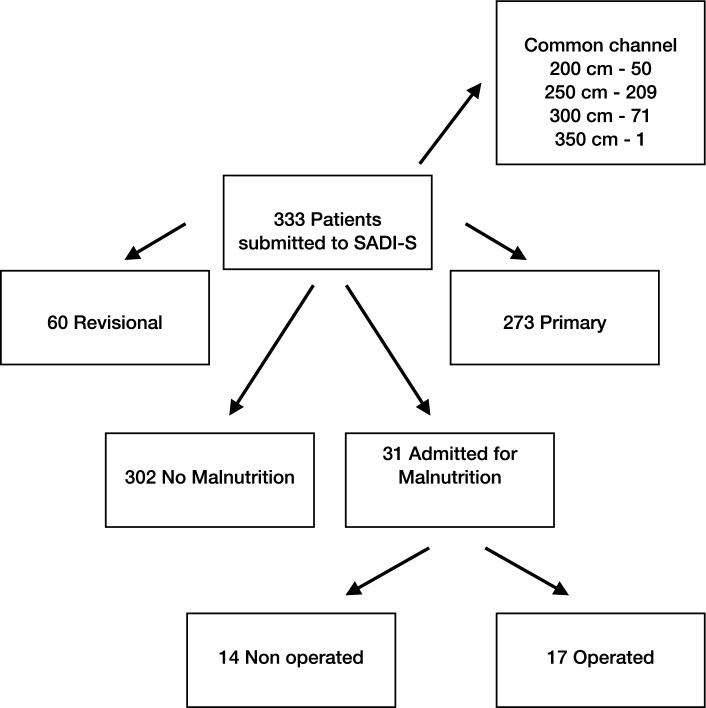
Flow chart

From May 2007 to February 2019, 333 consecutive patients (220 female) were submitted to SADI-S, either as a one-step surgery (273 cases) or as a second surgery after a previous bariatric operation (60 patients). The study was limited to patients operated before 2019 to warrant at least a 5 year follow up. The common limb was 200 cm in 50 cases, 250 cm in 211 cases, 300 cm in 71 and 350 in 1. Indications for SADI-S, surgical technique, supplementation and follow up have been reported previously [5]. The mean age was 47 years (21–71), mean weight 124 kg (61–216), and mean body mass index (BMI) 46.2 kg/m^2^ (24,7–76). One-hundred and sixty-one patients were diabetics, 58 under insulin treatment. Hypertension was present in 147 cases. Thirty-one patients (9,3%) were admitted for severe malnutrition. This was defined as low albumin levels plus edema, in the absence of an inflammation status, and not amenable for ambulatory management. Seventeen patients were submitted to revisional surgery (5,1%) and constitute the series of our study (Fig. 1, Flow Chart).

### Statistics

Data are expressed by mean, range and standard error of the mean in case of normal distribution and median plus inter-quartile rank for non-normal distributions. Comparisons between the study series and the total SADI-S series are done with the t-test or the Kruskal Wallis test for numerical variables, and the chi-square test for categorical ones. Log-rank curves are built for significant continuous variables, and the Youden Index was used to discriminate the cut-off value.

## Results

Follow up for the whole series was 85% at 5 years. Seventeen patients (5,1%) were reoperated. They were 6 men and 11 women, with a mean age at the index operation of 50,3 years (35 to 68), a mean initial weight of 118 kg (86 to 180), and a mean initial BMI of 44,1 kg/m^2^ (38–62). Type 2 diabetes was present in 11 patients, 8 under insulin treatment; 15 patients had hypertension (88%), 11 had dyslipidemia (69%), and 9 had sleep apnea (56%). One patient had been submitted to a previous bariatric operation, a sleeve gastrectomy. SADI-S had been performed with a 2-m common limb in 8 cases (8/50, 16% malnutrition rate for SADI-S 200), and with a 250 cm common limb in the other 9 patients (9/156, 4,3% malnutrition rate for SADI-S 250). No one had undergone SADI-S with a 3-m common limb.

### Presentation

The mean time to first admission was 29 months, 3 months being the earliest one and 180 months the latest one. The mean number of hospital admissions before revision was 2 (0–5). Seven patients (41%) had the first episode requiring hospital admission in the first postoperative year, while 4 of them (23,5%) presented with malnutrition more than 5 years from surgery. In all patients with severe malnutrition, triggering causes were investigated — erratic feeding, malabsorption of bile salts (testing with ^75^Selenium-Homocholic Acid Taurine Scintigraphy—SeHCAT) or Small Intestine Bacterial Overgrowth (SIBO). Most patients responded to dietary education and oral nutritional supplementation, treatment with resin-cholestyramine or antibiotics in cases of suspected SIBO.

The mean time to reoperation was 56 months, from 16 to 186 months; 50% of the patients were reoperated in the first 3 postoperative years, and 37% of them beyond the 5th postoperative year.

Indication for reoperation was individualized, and different parameters influenced this decision, as were age, severity of the episode, psychiatric evaluation, social status, number of episodes, coexisting diseases, etc. The main cause for reoperation was severe malnutrition in most cases (12 patients). The mean (SD) total protein value was 5.3 (0.76) gr/dL, and mean albumin 2.6 (0.6) gr/dL, and all patients presented with edema in lower limbs and moderate to severe deficiencies in fat-soluble vitamins and minerals (zinc, selenium, and copper). Intractable diarrhea was present in some patients (four cases); there was one patient with severe hypocalcemia and 3 had developed liver failure, two secondary to alcohol consumption and one to viral hepatitis and cirrhosis (Table 1).

**Table 1:** Indications for surgery, findings, and surgical attitude

Case nº	Limb length	Indication	Other causes	Time to surgery	Nº of admissions	Real length	Technique	Stomach
1	200	Diarrhea	Hypocalcemia	65	5	200	SADI-S 350	Re-sleeve
2	200	Malnutrition	Diarrhea	114	2	200	SADI-S 350	
3	200	Malnutrition		28	1	200	RnY DS	
4	200	Malnutrition	Diarrhea	120	1	200	SADI-S 300	
5	200	Malnutrition	Aged	28	2	200	RnY DS	
6	200	Liver failure	Alcohol	133	1	200	SADI-S 300	Re-sleeve
7	200	Diarrhea		37	1	250	SADI-S 350	
8	250	Malnutrition		156	1	170	SADI-S 300	
9	250	Malnutrition		17	2	250	RnY DS	
10	250	Malnutrition	Schizophrenia	16	1	250	Duodeno-duodenostomy	
11	250	Malnutrition		40	1	250	SADI-S 350	Re-sleeve
12	250	Liver failure	Viral hepatitis	23	3	220	Duodeno-jejunostomy	
13	250	Malnutrition		19	3	150	SADI-S 350	
14	250	Malnutrition		76	1	190	SADI-S 350	
15	250	Liver Failutre	Alcohol	16	1	250	SADI-S 350	
16	250	Malnutrition		20	1	200	SADI-S 300	
17	250	Malnutrition		50	3	230	SADI-S 320	

Bold values are those that could have a prognostic significance

Mean age at reoperation was 55 years (32v71), mean weight 57 kg (46–74), mean BMI 21 kg/m^2^ (18,6–27) and mean total weight loss (TWL) 50,2% (32,7–68). All malnourished patients were previously eligible for nutritional support, including total parenteral nutrition in 7 cases, until an adequate nutritional status was achieved before surgery. The mean number of daily bowel movements was 5,6 [2–10].

### Surgical findings

The common limb was measured in all cases. In patients with an initial SADI-S 200, in 6 cases, the measured length was 2 m; in 1 patient the limb was shorter, 170 cm, and in another one it was longer, 250 cm. In patients submitted to SADI-S 250, we found a correct common limb length in 4 cases, and a shorter one in the other 5 (230, 220, 200, 190 and 150 cm) (Table 1).

### Surgical technique

**Fig. 2:**
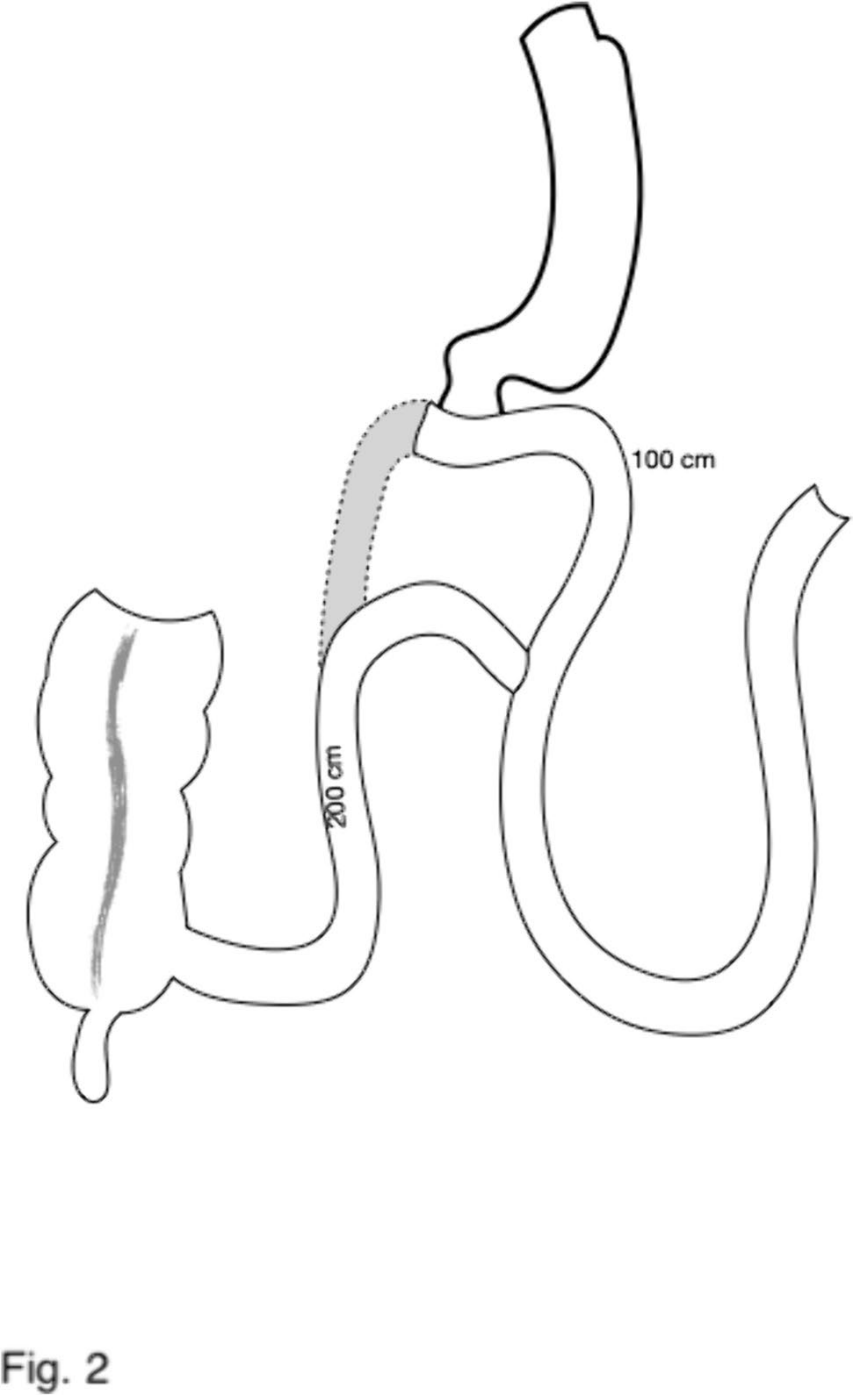
Scheme of the conversion of SADI-S to Roux en Y DS with an antiperistaltic alimentary limb. The efferent (common) limb has been divided close to the anastomosis and re-anastomosed to the afferent (biliopancreatic) limb 1 m proximal to the previous duodeno-ileostomy. The final operation has a 100 cm antiperistaltic alimentary limb and a 200 cm common limb

The first three patients had a conversion into a Roux en Y DS through a division of the efferent limb just beyond the anastomosis, and the creation of a new anastomosis between this and the afferent 1 m proximal to the duodeno-ileostomy (Fig. 2). In this way, the three patients had a DS with an antiperistaltic alimentary limb (1 m) and a 2-m common limb. Three patients were submitted to a “total” reversal (1 case, end to end duodeno-duodenostomy) or an “almost-total” reversal, a duodeno-jejunostomy in the first jejunal loop (two cases). The other 11 patients were submitted to an elongation of the common limb through the division of the previous anastomosis and the creation of a new one more proximally, leaving a final common channel of 270 in one case, 300 cm in 3, 320 in 1 and 350 cm in 7 cases. A resizing of the sleeve gastrectomy over a 54 French bougie was performed along with the proximalization of the SADI-S in 3 cases; all three were suffering from severe diarrhea with no or mild hypoalbuminemia and had a very enlarged stomach (Table 1).

### Outcome

After surgery, one patient with an advanced respiratory disease rejected ventilatory support and died. One patient with a Roux-en-Y DS was readmitted with an intestinal obstruction for an internal hernia, and was reoperated; intestinal resection was necessary, and she finally was converted into a Roux-en-Y Gastric Bypass. One patient with a Roux-en-Y DS with an antipersitaltic alimentary limb was reoperated 10 years later for gastro-ileal stasis with postprandial fullness and frequent regurgitation and vomiting. She was converted into a SADI-S with a 300 cm common limb.

### Long-term results

In the long term the mean weight was 71 kg (44–89) with a mean weight regain of 14 kg from revisional surgery; the mean BMI was 26 kg/m^2^ (17–37), and the stool frequency was normalized, with mean number of bowel movements of 1,3 (0,5–4).

### Factors related to malnutrition

Clinical and laboratory data of patients needing surgery for malnutrition were crossed with data from the complete series of patients submitted to SADI-S to find out which parameters could be related to the development of malnutrition. Results are presented in Table 2. The presence of hypertension, a low level of preoperative alkaline phosphatase, a higher level of aspartate aminotransferase (AST) and alanine aminotransferase (ALT), a shorter common limb and admission for undernutrition were all significantly related to the need of reoperation. In diabetics, a higher preoperative glycemia, a higher preoperative glycated hemoglobin (HbA1c) and the need of insulin therapy were related to malnutrition. A greater excess weight loss (EWL) in the first 6 and 12 months (EWL > 77% and EWL > 98%, respectively) was also related to severe malnutrition (area under the curve 0,771 and 0,763). No relationship was observed with the presence of steatosis in the pathologic study of the liver.

**Table 2:** Preoperative clinical and analytical data and relation to reoperation

	No malnutrition	Reoperation	*p* value	Cut off	Area under the curve
Male %	33%	35%	0,9		
Age (years)	46,8 (0,6)	50,2 (2,4)	0,09		
BMI (kg/m^2^)	46,3 (0,40)	44,1 (1,66)	0,09		
Hemoglobin (g/dL)	14,06 (0,08)	13,6 (0,37)	0,10		
Hematocrit (%)*	42,7 (4,7)	41,3 (5,47)	0,06		
Iron (µg/dL)*	72 (38)	60 (48,25)	0,24		
Albumin (g/dL)*	4,2 (0,4)	4,15 (0,37)	0,22		
Total proteins (g/dL)*	7,1 (0,5)	7,3 (0,45)	0,17		
Calcium (mg/dL)*	9,5 (0,6)	9,7 (0,53)	0,23		
Glucose (mg/dL)	157,2 (4,7)	203,3 (20,3)	**0,08**	** ≥ 155**	**0,721**
Glycated hemoglobin (%)	7,30 (0,12)	8,83 (0,69)	**0,001**	** ≥ 6,65**	**0,716**
Triglycerides (mg/dL)*	130 (90)	155 (125,5)	0,3		
Total cholesterol (mg/dL)*	185 (52)	193 (71,25)	0,31		
AST (U/L)*	22 (10)	23 (15,7)	**0,05**	** ≥ 18,5**	**0,639**
ALT (U/L)*	24 (17)	30 (23,5)	**0,007**	** ≥ 29,5**	**0,697**
GGT (U/L)*	44 (4,02)	74,5 (36,08)	0,11		
Alkaline phosphatase (U/L)*	78 (28)	61,5 (32,5)	**0,04**	** ≤ 60,5**	**0,681**
T2DM	47%	64%	0,51		
Hypertension	42%	88%	**0,003**		

Values are given in Mean (Standard error of the mean) or * Median (Interquartile range). The cut-off is the value from which the possibility of a revisional operation was found to be significantly greater based on the Receiver Operating Curve (ROC) and the Youden Index

## Discussion

SADI-S is a safe operation for selected patients with adequate follow up and life-long supplementation [4]. However, despite being apparently safer than prior malabsorptive operations as BPD and DS, some patients can experience malnutrition, a life-threatening condition that must be avoided or early detected and treated [5].

The main findings of this review of our experience are that a short common channel (200 cm) is followed by a high rate of severe malnutrition; that errors in the measurement of the small bowel may also lead to malnutrition; and that there are some patients who can be especially vulnerable, as those with hypertension and poor-controlled type-2 diabetes and patients with alterations in the liver tests. Clinicians must be aware when patients lose weight too rapidly, have more severe diarrhea, or their laboratory tests are significantly altered in the first postoperative year, as they are all markers of malnutrition.

The initial operation with a common channel of 2 m was abandoned in 2009 because of the high incidence of malnutrition [2]. The simple increase in 50 cm did not affect the good results on weight loss, and, on the other hand, increased the safety of the operation [5]. To achieve a secure technique, the measurement of the ileum must be accurate enough, either if it is done with marked graspers or if it is performed with a tape of known length [6, 7]. In all the patients included, measurement of the bowel in the first operation had been performed with a grasper marked at 10 cm from the tip. We also recommend measuring the bowel after relaxing the smooth muscle with intravenous Buscopan®, as the stretching of the small bowel has demonstrated to obtain more accurate measurements [8].

Seventy-five percent of the patients had severe protein malnutrition, according to screening questionnaires [Malnutrition Universal Screening Tool (MUST), Nutritional Risk Screening-2002 (NRS-2000) or The Global Leadership Initiative on Malnutrition (GLIM)] [9], and confirmation by low levels of serum proteins and edema, in absence of inflammation (C-reactive protein < 2 mg/dL). Furthermore, patients presented symptoms of muscular weakness suggestive of sarcopenia. Inflammation is a relevant issue for the correct diagnosis of malnutrition as its presence detracts significance to albumin levels [10].

Some bariatric surgery studies have evaluated this clinical condition using the Controlling Nutritional Status (CONUT) score, where malnutrition parameters (serum albumin and cholesterol) are combined with immunologic parameters (total lymphocyte count), to provide additional information on malnutrition related to disease [11].

We did not find a significant association between older age and malnutrition. Cossu et al. [12] reviewed the importance of age on malnutrition in patients submitted to BPD, finding that the group of patients older than 55 years had a 16% rate of malnutrition and an 8% rate of revisional surgery. The authors outlined the difficulty of aged patients in changing alimentary habits after surgery and the clinical importance of some disorders related to aging, as it is loss of appetite, the impaired sense of taste, the lack of teeth and therefore good mastication and sometimes even depression.

We found that hypertension was more prevalent between reoperated patients, as well as higher preoperative values of glycemia and HbA1c. Hypertension and poor control of diabetes, defined by HbA1c above 7% or high glycemia are both related to atherosclerosis, which along with age could be the cause of an impaired intestinal absorption and a decreased adaption capacity of the small bowel. Although there are no studies on microvascularization of the small bowel and nutrition, atherosclerosis has been previously related to malnutrition in patients with peripheral arterial disease [13].

We find no clear explanation to the relationship between a low preoperative alkaline phosphatase (AP) and malnutrition; low AP levels are related to low zinc and magnesium levels, what is seen in malnutrition. Unfortunately, we do not routinely analyze them preoperatively, so we cannot establish a connection between them. Although we didn’t find a correlation between the liver biopsy and malnutrition, alteration of liver tests, AP, AST and ALT, were all related to malnutrition. Liver disease, obesity, weight loss and malnutrition are intimately related. Non-alcoholic fatty liver disease (NAFL) improves after a successful weight loss operation. However, when weight loss happens too fast, the loss of adipose tissue leads to a massive mobilization of free fatty acids that reach the liver and provoke hepatotoxicity. This is compensated by the reduction of liver fat caused by the weight loss [14]. But when other mechanisms are present, as protein malnutrition or deficiency of specific amino acids, or bacterial overgrowth, this toxicity can lead to a liver failure. Bacterial overgrowth is not supposed to happen after SADI-S, because there is not a real blind loop, as it was in old malabsorptive operations. Nevertheless, the mere absence of nutrients in an intestinal loop is enough to induce a substantial change in the jejunal microbiota [15], and this can have dramatic consequences in patients with liver disease. Patients with an underlying liver disease should never be offered this type of surgery because of the risk of hepatic insufficiency [16] that on some occasion has led to the need of liver transplantation [17].

Surgeons and endocrinologists should be specially alerted when weight loss is too fast in the first postoperative year, or when there is a high number of bowel movements. These patients have to be tested for micronutrient or protein insufficiency before clinical manifestations and treated with supplementation of protein and vitamins.

Regarding the revisional technique, we initially introduced the Roux-en-Y configuration with the antiperistaltic alimentary limb to avoid touching the previous duodeno-ileostomy. When we demonstrated the feasibility of dividing the anastomosis and performing a new duodeno-enterostomy without affecting the pylorus function, the initial technique was abandoned. Certainly, its results were not very good. The opening of the mesentery favored an internal hernia in one case, and the antipersitaltic Roux limb jeopardized gastric emptying and originated severe reflux and regurgitation in another one. As a rule we first measure the biliary and the common limb, to know if the cause of malnutrition was an error of the first operation, and to assess how long can the proximalization be done. Then, 100 to 150 cm are usually added to the common limb, placing the new anastomosis at 300 to 350 cm from the ilio-cecal junction. When there is a severe underlying disease, as happened with the psychiatric patient and with those suffering from liver failure, the new anastomosis is placed in the first jejunal loop or even in the duodenum, restoring an almost normal anatomy.

In summary, SADI-S is an effective and safe operation when the selection of patients, the performance of the operation and the supplementation and follow up are correct. Older patients, patients with hypertension, lower BMI, or poor-controlled diabetes, as well as those with severe accompanying conditions or those with problems of adherence to supplementation and follow up should not undergo SADI-S 250. Limitations of this study are the single institution experience, the retrospective nature of the study, the short number of patients and the heterogeneity of the series.

## Ethics statement

For this type of study, formal consent is not required. The authors declare that they have no conflicts of interest regarding this manuscript.

## Data Availability

The data that support the findings of this study are not openly available due to local data protection restrictions but are available from the corresponding author upon reasonable request.

## References

[1] Sánchez-Pernaute A, Rubio Herrera MA, Pérez-Aguirre E, García Pérez JC, Cabrerizo L, Díez Valladares L, Fernández C, Talavera P, Torres A (2007) Proximal duodenal-ideal end-to-side bypass with sleeve gastrectomy: proposed technique. Obes Surg 17:1614–1618. 10.1007/s11695-007-9287-8 18040751 10.1007/s11695-007-9287-8

[2] Sánchez-Pernaute A, Rubio MA, Pérez Aguirre E, Barabash A, Cabrerizo L, Torres A (2013) Single-anastomosis duodenoileal bypass with sleeve gastrectomy: metabolic improvement and weight loss in first 100 patients. Surg Obes Relat Dis 9:731–735. 10.1016/j.soard.2012.07.018 22963820 10.1016/j.soard.2012.07.018

[3] Gagner M (2016) Hypoabsorption not malabsorption, hypoabsorptive surgery and not malabsorptive surgery. Obes Surg 26:2783–2784. 10.1007/s11695-016-2350-6 27573648 10.1007/s11695-016-2350-6

[4] Vilallonga R, Balibrea JM, Curell A, Gonzalez O, Caubet E, Ciudin A, Ortiz-Zúñiga AM, Fort JM (2017) Technical options for malabsorption issues after single anastomosis duodenoileal bypass with sleeve gastrectomy. Obes Surg 27:3344–3348. 10.1007/s11695-017-2931-z 28952026 10.1007/s11695-017-2931-z

[5] Sánchez-Pernaute A, Rubio Herrera MA, Pérez Ferré N, Sáez Rodríguez C, Marcuello C, Pañella C, López Antoñanzas L, Torres A, Pérez-Aguirre E (2022) Long-term results of single-anastomosis duodeno-ileal bypass with sleeve gastrectomy (SADI-S). Obes Surg 32:682–689. 10.1007/s11695-021-05879-9 35032311 10.1007/s11695-021-05879-9 PMC8760573

[6] Wagner M, Mayer BFB, Bodenstedt S, Kowalewski K, Nickel F, Speidel S, Fischer L, Kenngott HG, Müller-Stich B (2021) Comparison of conventional methods for bowel length measurement in laparoscopic surgery to a novel computer-assisted 3D measurement system. Obes Surg 31:4692–4700. 10.1007/s11695-021-05620-6 34331186 10.1007/s11695-021-05620-6 PMC8490232

[7] Slagter N, van Wilsum M, de Heide LJM, Jutte EH, Kaiser MA, Damen SL, van Beek AP, Emous M (2022) Laparoscopic small vowel length measurement in bariatric surgery using a hand-over-hand technique with marked graspers: an ex vivo experiment. Obes Surg 32:1201–1208. 10.1007/s11695-022-05918-z 35201571 10.1007/s11695-022-05918-z PMC8933352

[8] Karagül S, Kayaalp C (2019) Repeated stretched or non-stretched small bowel length measurements in healthy individuals. Turk J Surg 35:1–5. 10.5152/turkjsurg.2018.4087 32550296 10.5578/turkjsurg.4087 PMC6791677

[9] Cederholm T, Jensen GL, Correia MITD, Gonzalez MC, Fukushima R, Higashiguchi T, Baptista G, Barazzoni R, Blaauw R, Coats AJS, Crivelli AN, Evans DC, Gramlich L, Fuchs-Tarlovsky V, Keller H, Llido L, Malone A, Mogensen KM, Morley JE, Muscaritoli M, Nyulasi I, Pirlich M, Pisprasert V, de van der Schueren MAE, Siltharm S, Singer P, Tappenden K, Velasco N, Waitzberg D, Yamwong P, Yu J, Van Gossum A, Compher C, GLIM Core Leadership Committee, GLIM Working Group (2019) GLIM criteria for the diagnosis of malnutrition - A consensus report from the global clinical nutrition community. J Cachexia Sarcopenia Muscle 10:207–217. 10.1002/jcsm.12383 30920778 10.1002/jcsm.12383 PMC6438340

[10] Cederholm T, Jensen GL, Ballesteros-Pomar MD, Blaauw R, Correia MITD, Cuerda C, Evans DC, Fukushima R, Ochoa Gautier JB, Gonzalez MC, van Gossum A, Gramlich L, Hartono J, Heymsfield SB, Jager-Wittenaar H, Jayatissa R, Keller H, Malone A, Manzanares W, McMahon MM, Mendez Y, Mogensen KM, Mori N, Muscaritoli M, Nogales GC, Nyulasi I, Phillips W, Pirlich M, Pisprasert V, Rothenberg E, de van der Schueren M, Shi HP, Steiber A, Winkler MF, Barazzoni R, Compher C (2023) Guidance for assessment of the inflammation etiologic criterion for the GLIM diagnosis of malnutrition: a modified Delphi approach. Clin Nutr 43:1025–1032. 10.1016/j.clnu.2023.11.026 38238189 10.1016/j.clnu.2023.11.026

[11] Gentileschi P, Siragusa L, Alicata F, Campanelli M, Bellantone C, Musca T, Bianciardi E, Arcudi C, Benavoli D, Sensi B (2022) Nutritional Status after Roux-En-Y (Rygb) and One Anastomosis Gastric Bypass (Oagb) at 6-Month Follow-Up: A Comparative Study. Nutrients 14:2823. 10.3390/nu14142823 35889780 10.3390/nu14142823 PMC9324253

[12] Cossu ML, Fais E, Meloni GB, Profili S, Masala A, Alagna S, Rovasio PP, Spartà C, Pilo L, Tilocca PL, Noya G (2004) Impact of age on long-term complications after biliopancreatic diversion. Obes Surg 14:1182–1186. 10.1381/0960892042387093 15527631 10.1381/0960892042387093

[13] Carvalho J, Correia MA, Kanegusuku H, Longano P, Wolosker N, Ritti-Dias RM, Cucato GG (2022) Association between the risk of malnutrition and functional capacity in patients with peripheral arterial disease: a cross-sectional study. PLoS ONE 17:e0273051. 10.1371/journal.pone.0273051 36083948 10.1371/journal.pone.0273051 PMC9462727

[14] Vespasiani-Gentilucci U, Vorini F, Carotti S, De Vincentis A, Galati G, Gallo P, Scopinaro N, Picardi A (2017) Hepatic complications of bariatric surgery: the reverse side of the coin. Acta Gastroenterol Belg 80:505–513 29560647

[15] Prakash G, Drenick EJ, Wexler H, Delucia L, Finegold SM (2018) Microbial flora in the bypassed jejunum of patients with biliopancreatic bypass for obesity. Am J Clin Nutr 46:273–276 10.1093/ajcn/46.2.273 3618530

[16] Kirkpatrick V, Moon RC, Teixeira AF, Jawad MA (2018) Cirrhosis following single anastomosis duodeno-ileal switch: a case report. Int J Surg Case Rep 45:130–132. 10.1093/ajcn/46.2.273 29605778 10.1016/j.ijscr.2018.03.021 PMC6000903

[17] Addeo P, Cesaretti M, Anty R, Iannelli A (2019) Liver transplantation for bariatric surgery-related liver failure: a systematic review of a rare condition. Surg Obes Relat Dis 15:1394–1401. 10.1016/j.soard.2019.06.002 31285130 10.1016/j.soard.2019.06.002

